# Synthesis of Fe_3_O_4_@Au Core–Shell Nanoparticles with Varying Thicknesses for Application in Computed Tomography Imaging

**DOI:** 10.1002/open.202500166

**Published:** 2025-04-21

**Authors:** Nguyen Thi Ngoc Linh, Nguyen Hoa Du, Ngo Thanh Dung, Le Thi Thanh Tam, Pham Hong Nam, Phan Thi Hong Tuyet, Le Trong Lu, Le The Tam

**Affiliations:** ^1^ Faculty of chemistry Thai Nguyen University of Sciences Thai Nguyen University Tan Thinh Ward Thai Nguyen City 123 Vietnam; ^2^ School of Chemistry Biology and Environment Vinh University 182 Le Duan Vinh City Nghean 123 Vietnam; ^3^ Institute of Materials Science Vietnam Academy of Science and Technology 18 Hoang Quoc Viet Road Hanoi 123 Vietnam; ^4^ Graduate University of Science and Technology Vietnam Academy of Science and Technology 18 Hoang Quoc Viet Road Hanoi 123 Vietnam

**Keywords:** computed tomographies, core–shells, Fe_3_O_4_@Au, hybrid nanoparticles

## Abstract

To enhance the resolution of medical images, core–shell Fe_3_O_4_@Au hybrid nanoparticles (HNPs) with gold shells of varying thicknesses are developed. By optimizing synthesis conditions, nanoparticles with uniform size, clear core‐shell structure, and high stability in biological environments are obtained. In vitro testing data shows that HNPs with a 5–7 nm‐thick gold shell exhibits a high X‐ray attenuation. The in vivo efficacy of the Fe_3_O_4_@Au HNPs is tested in liver and kidney tissues in a mouse model for study on drug kinetics. Results demonstrate the effectiveness of Fe_3_O_4_@Au HNPs in enhancing the contrast of computed tomography (CT) images, especially in the liver. Simultaneously, the clearance process of nanoparticles through the kidneys is also observed, opening up the prospect of applying these nanoparticles in image diagnosis and treatment monitoring. Overall, these hybrid particles are a promising candidate for CT imaging techniques.

## Graphical abstract

To enhance resolution of medical images, core–shell Fe_3_O_4_@Au hybrid nanoparticles (HNPs) with gold shells of varying thicknesses are developed. In vitro testing data shows that HNPs with a 5–7 nm‐thick gold shell exhibits high X‐ray attenuation. The in vivo efficacy of HNPs is tested in liver and kidney tissues in a mouse model for study on drug kinetics.

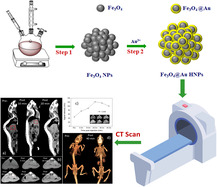

## 1 Introduction

Computed tomography (CT) has become an essential diagnostic imaging tool in modern medicines, thanks to its noninvasive imaging technique. CT can produce high‐resolution images and rapid scan times, providing accurate anatomical information in the diagnosis and monitoring of various diseases.^[^ ^1^, ^2^ ^]^ The operating principle of CT is based on the differences in X‐ray attenuation of multiple tissues. Tissues with different electron densities will attenuate the X‐ray beam to varying degrees. Computers process the data collected from detectors to reconstruct 3D images of specific tissues and organs of the body.^[^ ^3^, ^4^ ^]^ The attenuation coefficient, which characterizes the X‐ray absorption capability of a tissue, depends on the atomic number and electron density of that tissue. Currently, the primary Food and Drug Administration‐approved intravenous contrast agents for CT imaging are iodinated small molecules such as ioversol, iomeprol, iodixanol, or iohexol, which are widely used to enhance image quality and contrast.^[^ ^5^, ^6^ ^]^ These agents, characterized by high X‐ray attenuation, significantly improve the resolution of images of anatomical structures, helping physicians easily differentiate between diseased and healthy tissues. However, these contrast agents have a short blood circulation time and are rapidly excreted by the kidneys.^[^ ^7^ ^]^ Moreover, their use carries a high risk of adverse reactions, including allergic reactions and acute kidney injury, especially in patients with impaired renal function.^[^ ^8^ ^]^ Recent research has focused on developing gold nanoparticle (NP) systems as a new generation of contrast agents for CT to overcome the limitations of iodinated contrast agents.^[^ ^9^, ^10^, ^11^ ^]^ The density and atomic number of gold are 19.32 g cm^−3^ and 79, respectively, significantly higher than those of iodine currently in use (4.9 g cm^−3^ and 53, respectively). The photon attenuation coefficient of gold and iodine at 100 keV is 5.16 and 1.94, respectively. Hence, the gold NPs possess superior X‐ray absorption capability compared to iodine, leading to higher image contrast.^[^ ^12^ ^]^ Additionally, Au has chemical inertness, high biocompatibility, longer blood circulation time, and easy surface functionalization, opening up numerous prospects for applying gold NPs in CT imaging. However, the high production cost is a barrier to the widespread clinical application of gold NPs. Fe_3_O_4_ NPs exhibit superparamagnetism, low toxicity, high biocompatibility, and a lower price than Au NPs. Combining Fe_3_O_4_ and Au components on a hybrid nanostructure is a solution to replace single Au NPs.^[^ ^13^ ^]^ In particular, the magnetic properties of Fe_3_O_4_ integrated into hybrid NPs allow for precise control and localization of NPs at the region of interest (ROI) using an external magnetic field,^[^ ^14^ ^]^ promising to reduce the contrast agent dose and increased diagnostic accuracy. The Fe_3_O_4_@Au hybrid nanostructure with strong X‐ray interaction of the Au shell has opened up a promising new direction in CT imaging.^[^ ^15^, ^16^ ^]^ However, optimizing the structure and properties of Fe_3_O_4_@Au HNPs, especially the relationship between the thickness of the Au shell and the enhancement of CT image contrast, is still an issue that needs further investigation to bring this material into clinical application.

This article reports a synthesis process for core–shell Fe_3_O_4_@Au HNPs in an organic solvent, using small, monodispersed Fe_3_O_4_ NPs as seeds. By adjusting the concentration of the HAuCl_4_ precursor, we obtained Fe_3_O_4_@Au HNPs with varying thicknesses of the Au shell. The synthesized hybrid NPs were phase transferred using the polymer poly(maleic anhydride‐alt‐1‐octadecene) (PMAO) to enhance biocompatibility and dispersibility in aqueous media. With their unique core–shell structure, Fe_3_O_4_@Au HNPs have a strong X‐ray absorption capacity due to the Au shell, which will enhance the signal intensity in CT imaging. This study focuses on the relationship between the thickness of the gold shell in Fe_3_O_4_@Au HNPs and the X‐ray attenuation efficiency based on in vitro and in vivo experiments.

## 2 Results and Discussion

### 2.1 The Formation of Fe_3_O_4_ NPs

**Figure 1:**
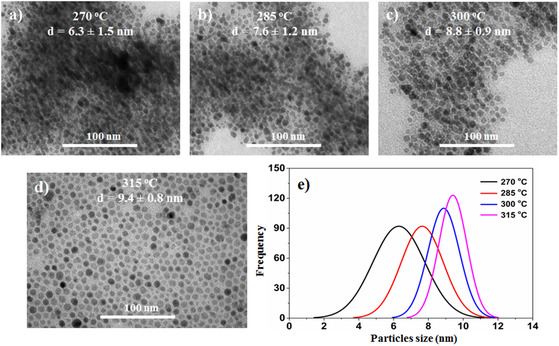
a–d) TEM images and e) the particle size distribution of the nano‐Fe_3_O_4_ fabricated at different temperature.

The fabrication of the hybrid structure materials between Fe_3_O_4_ and Au for the biological application takes two steps as depicted in Scheme S1, Supporting Information. The effect of reaction temperature on the morphology of Fe_3_O_4_ NPs was investigated in the range of 270–315 °C. **Figure** **1** shows the transmission electron microscopy (TEM) images and particle size distribution plots. Results showed that samples synthesized at 270 °C began to exhibit nonuniform Fe_3_O_4_ NPs with unclear boundaries, with an average particle size of 6.3 ± 1.5 nm, and there were still some NP clusters (Figure 1a). Samples prepared at 285 °C and 300 °C yielded nearly spherical, nonuniform Fe_3_O_4_ NPs and clearer boundaries than at 270 °C. The average particle sizes of these two samples were 7.6 ± 1.2 nm and 8.8 ± 0.9 nm, respectively, with particle size deviations of 15.8% and 10.2% (Figure 1b,c). At 315 °C (corresponding to the boiling point of the 1‐octadecene (ODE) solvent), the obtained Fe_3_O_4_ NPs were relatively uniform with clear boundaries and an average particle size of 9.4 ± 0.8 nm, with a particle size deviation of 8.5%. Subsequently, the reaction temperature significantly affects the formation and growth of Fe_3_O_4_ NPs. Below the boiling point of the solvent (below 315 °C), the iron intermediate complexes (oleate complexes) are not completely decomposed, leading to incompletely formed NPs (especially at 270 °C). Above 315 °C, the decomposition of iron intermediate complexes completes, forming Fe_3_O_4_ NPs with a uniform particle size and clear boundaries. At higher the reaction temperature, the thermal motion of the subparticles is stronger. Consequently, the coalescence of subparticles reduces their surface energy (Ostwald ripening effect),^[^ ^17^ ^]^ producing Fe_3_O_4_ NPs with larger particle sizes. Thus, at 315 °C, a highly uniform material is obtained, which was an appropriate seed for Fe_3_O_4_@Au nanostructures.

### 2.2 The Formation of Fe_3_O_4_@Au HNPs

**Figure 2:**
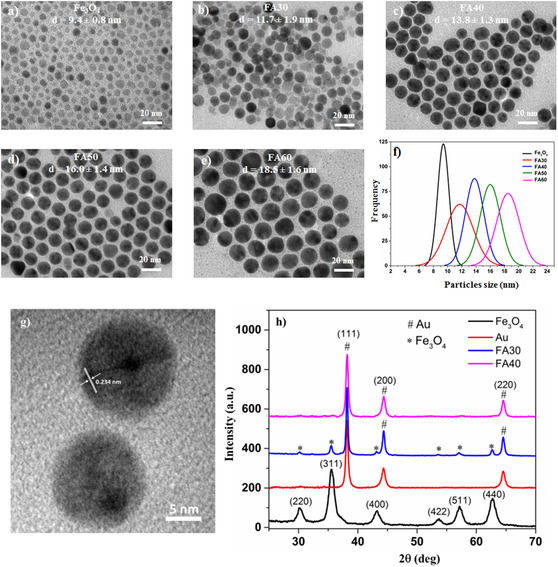
a–e) TEM images and f) particle size distribution of Fe_3_O_4_ NPs and Fe_3_O_4_@Au HNPs synthesized with different concentrations of HAuCl4, g) HRTEM image of Fe_3_O_4_@Au HNPs (sample FA40), and XRD patterns of Fe_3_O_4_ NPs, Au NPs, and h) Fe_3_O_4_@Au HNPs.

In this study, we investigated the influence of HAuCl_4_ precursor concentration on the formation and development of Fe_3_O_4_@Au hybrid nanostructures. **Figure** **2** presents TEM images and size distribution plots of Fe_3_O_4_ seeds (Figure 2a) and Fe_3_O_4_@Au HNPs synthesized with HAuCl_4_ concentrations ranging from 30 to 60 mM (Figure 2b–e). The morphology and size of the Fe_3_O_4_@Au HNPs depended on the concentration of the HAuCl_4_ precursor used in the experiments. When the HAuCl_4_ concentration was 30 mM (sample FA30), nonuniform NPs were obtained, including larger dark‐colored spherical particles and smaller lighter‐colored particles. The larger spheres were Fe_3_O_4_@Au HNPs with a Fe_3_O_4_ core and an Au shell (core–shell structure). The smaller spheres were Fe_3_O_4_ NPs that were either uncoated or coated with a lean layer of Au, resulting in sizes similar to the Fe_3_O_4_ seeds. The nonuniform thickness of the Au shell could be due to the low concentration of the HAuCl_4_ precursor used. In this case, the average particle size was 11.7 ± 1.9 nm, with a large size deviation of 16.2%. When the HAuCl_4_ concentration increased to 40 mM (sample FA40), there were no free Fe_3_O_4_ NPs, and the Fe_3_O_4_@Au core–shell structures obtained were relatively uniform in shape and size. In this case, the average particle size of the hybrid particles was 13.8 ± 1.3 nm, with a small size deviation of 9.4%. As the HAuCl_4_ concentration raised to 50 and 60 mM (samples FA50 and FA60), the Fe_3_O_4_@Au HNPs maintained a uniform spherical shape as the 40 mM sample. However, their average sizes increased significantly to 16.0 ± 1.4 nm and 18.5 ± 1.6 nm for samples 50 and 60 mM, respectively, with size deviations in these cases being less than 10%. Thus, under the investigated conditions, as the HAuCl_4_ concentration increased from 40 to 60 mM, the obtained Fe_3_O_4_@Au HNPs were relatively uniform, with the average size rising from 13.8 to 18.5 nm (Figure 2f), corresponding to an increase in the thickness of the Au shell from 4.4 to 9.1 nm. The obtained results are in complete agreement with the predictions of the La Mer model.^[^ ^18^ ^]^ In the reaction system, the reduction of Au^3+^ to Au occurs, and the initially formed Au atoms deposit on the surface of the Fe_3_O_4_ seeds. The presence of Au atoms on the Fe_3_O_4_ magnetic core activates these sites, creating conditions for a large quantity of subsequent Au atoms to deposit. By the process, the Au shell gradually forms due to the deposition and growth of Au nanocrystals. However, when the concentration of the HAuCl_4_ precursor is low (sample FA30), the nonuniform distribution of Au atoms on the magnetic core facilitates the Ostwald ripening effect, reducing the deposition rate in regions with low‐Au atom density. Following that is the growth of the nonuniform Au shell. Therefore, sample FA30 contains Fe_3_O_4_@Au HNPs with varying thicknesses of the Au shell, from thick Au layers to very thin or no Au layers. When the concentration of the HAuCl_4_ precursor increases (samples FA40, FA50, and FA60), the generated Au source is large enough to prevent the Ostwald ripening process, creating conditions for the uniform growth of the Au shell on the Fe_3_O_4_ core and forming a relatively uniform Fe_3_O_4_@Au core–shell structure.

The interplanar spacing (*d*) was determined using high‐esolution transmission electronmicroscopy (HRTEM) to determine the crystal structure of the synthesized Fe_3_O_4_@Au HNPs. From the HRTEM image of sample FA40 (Figure 2g), a lattice fringe of 0.234 nm corresponding to the (111) plane of face‐centered‐cubic (fcc) Au (JCPDS No. 04‐0784) can be observed.^[^ ^19^ ^]^

To determine the crystal phase structure of the Fe_3_O_4_@Au HNPs, X‐ray diffraction (XRD) was used with a 2*θ* scanning angle from 25 to 70° (Figure 2h). The XRD data showed that the synthesized material had good crystallinity. The observed diffraction peaks at 2*θ* angles of 30.16, 35.49, 43.01, 53.78, 57.21, and 62.73° correspond to the (220), (311), (400), (422), (511), and (440) planes, respectively, characteristic of the inverse spinel fcc structure of Fe_3_O_4_ (JCPDS No. 19‐0629).^[^ ^20^ ^]^ The XRD pattern of the Au NPs showed diffraction peaks at 2*θ* angles of 38.07°, 44.21°, and 64.41°, corresponding to the (111), (200), and (220) planes, confirming the fcc structure of Au NPs (JCPDS No. 04‐0784).^[^ ^19^ ^]^ For the FA30 sample, high‐intensity diffraction peaks were at 2*θ* angles of 38.07°, 44.21°, and 64.41°, characteristic of the crystal structure of Au NPs, along with very‐low‐intensity diffraction peaks characteristic of Fe_3_O_4_ at 2*θ* angles of 30.16°, 35.49°, 43.01°, 57.21°, and 62.73°, with the peak at 53.78° almost completely disappearing. When the HAuCl_4_ concentration increased, the XRD pattern only showed the appearance of strong diffraction peaks characteristic of the Au crystal, and no characteristic peaks of the Fe_3_O_4_ crystal were observed, as shown in Figure 2h for sample FA40. Reasonably, the Fe_3_O_4_ core was completely coated by the Au shell (as observed in the TEM images in Figure 2c–e), causing the XRD signals of the Fe_3_O_4_ core to be shielded by the Au layer. This result is also consistent with the publications of some previous groups on the crystal structure of Fe_3_O_4_@Au core–shell HNPs.^[^ ^21^, ^22^ ^]^

**Figure 3:**
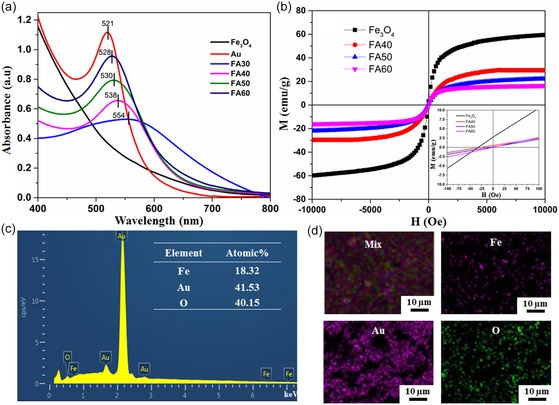
a) UV–vis spectra of Fe_3_O_4_ NPs, Au NPs, and Fe_3_O_4_@Au HNPs, b) magnetization curve of Fe_3_O_4_ NPs and Fe_3_O_4_@Au HNPs (inset shows the enlarged hysteresis loop at low magnetic field), and c) EDX spectrum and elemental atomic percentage of the sample, and EDX elemental mapping of sample FA60 (d).

The UV–vis absorption spectroscopy shown in **Figure** **3**a confirmed the formation of the Fe_3_O_4_@Au HNPs. In the wavelength range from 400 to 800 nm, Fe_3_O_4_ NPs did not exhibit an absorption maximum to indicate the surface plasmon resonance (SPR) effect or other optical mechanisms. Au NPs exhibited a SPR peak at 521 nm, with relatively narrow full width at half maximum of the plasmon absorption peak. The Fe_3_O_4_@Au HNPs exhibited SPR maxima in the wavelength range of 528–554 nm. The formation of the hybrid structure between Au and Fe_3_O_4_ led to a significant redshift and broadening of the SPR peak compared to Au NPs, with the degree of change depending on the concentration of the HAuCl_4_ precursor. The enhancement of the SPR properties of the HNPs can be explained by the change in the surface electronic properties of the Fe_3_O_4_ and Au components in the Fe_3_O_4_@Au HNPs. Studies have shown that Au NPs are electron rich, while magnetic Fe_3_O_4_ NPs are electron deficient. Therefore, when the Fe_3_O_4_@Au hybrid nanostructure is formed, electrons on the Au shell will transfer to the Fe_3_O_4_ core, causing the surface of the Au shell to become electron deficient.^[^ ^23^ ^]^ Yang's research group demonstrated that when the surface of noble metal NPs is electron rich, the SPR absorption maximum shifts to blue. Inversely, the electron deficiency causes the SPR position to shift to a longer wavelength.^[^ ^24^ ^]^ The optical properties of the Fe_3_O_4_@Au core–shell HNPs in this study are consistent with the hybrid model of Mie theory.^[^ ^25^, ^26^ ^]^ Moreover, the appearance of the SPR band in the UV–vis spectrum of the Fe_3_O_4_@Au nanostructure also confirms the formation of the Au shell on the Fe_3_O_4_ core, which is in complete agreement with the results observed from TEM images.

The magnetic properties of the materials were analyzed using a vibrating sample magnetometer at a magnetic field *H* = 10 kOe at 300 K, as shown in Figure 3b. The results showed that the saturation magnetization decreased from 60 emu g^−1^ (Fe_3_O_4_ sample) to 16.5 emu g^−1^ (FA60 sample). Thus, the saturation magnetization value of the lowest HNPs decreased by about 3.6 times compared to the Fe_3_O_4_ seeds. Analysis of the magnetization curve in the small magnetic field region from –100 to 100 Oe (inset in Figure 3b) showed that the coercivity *H*_c_ of the Fe_3_O_4_@Au HNPs also decreased significantly compared to the Fe_3_O_4_ seeds (38 Oe), with samples FA50 and FA60 having *H*_c_ = 0 Oe. Thus, this forms a sufficiently thick Au shell on the surface of the Fe_3_O_4_ NPs causing the material to become completely superparamagnetic at the test temperature (300 K). Since the magnetization of the NPs is in emu g^−1^, the decrease in the saturation magnetization *M*_s_ and coercivity *H*c of the Fe_3_O_4_@Au HNPs compared to the Fe_3_O_4_ seeds is due to the contribution of the nonmagnetic Au shell in the hybrid structure. In addition, the Fe_3_O_4_ seeds in all the hybrid samples have the same size, so when the HAuCl_4_ concentration in the hybrid sample increases, it will increase the thickness of the Au shell of the HNPs or the content of the nonmagnetic component (Au) increases, leading to a decrease in their saturation magnetization *M*_s_. Although the saturation magnetization *M*_s_ value of the HNPs is lower than that of the Fe_3_O_4_ seeds, their magnetic response capability is still relatively good. Thus, they are suitable for biomedical applications. Moreover, the energy‐dispersive X‐ray spectral (EDX) analysis of the representative sample FAu60 further confirmed the presence of Fe, Au, and O elements in Fe_3_O_4_@Au HNPs with atomic percentages of 18.32, 41.53, and 40.15%, respectively (Figure 3c). From the elemental mapping analysis (Figure 3d), the elements of the Fe_3_O_4_@Au HNPs are distributed evenly in the sample whose surface layer was mainly composed of the Au element. This result has demonstrated that the Fe_3_O_4_@Au HNPs with a core–shell structure have been successfully fabricated.

### 2.3 Stability and Particle Size of the PMAO‐Coated Fe_3_O_4_@Au HNPs

**Figure 4:**
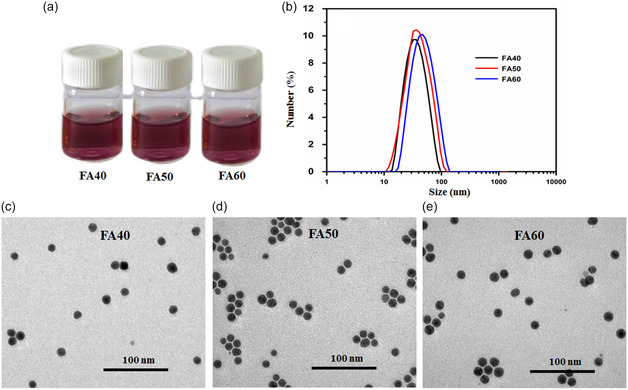
a) Images of the solution, b) hydrodynamic size, and c–e) TEM images of samples FA40, FA50, and FA60 after phase transfer using PMAO.

Throughout the synthesis, sodium oleate (SOA) acts as a complexing agent and a surfactant. Therefore, after fabrication, the surface of the Fe_3_O_4_@Au HNPs is coated with a layer of oleate, making them dispersible only in nonpolar solvents.^[^ ^27^ ^]^ For biomedical applications, the NPs must be dispersible in aqueous media. Therefore, in this study, samples FA40, FA50, and FA60 with different Au shell thicknesses were phase transferred and surface functionalized using the polymer PMAO. Here, PMAO is a molecule composed of both hydrophobic and hydrophilic parts. The hydrophobic part is a hydrocarbon group forming a bond with the hydrocarbon group of the oleate on the NP surface in the phase transfer process. The hydrophilic part is an anhydride group hydrolyzed to form COO^−^ groups due to the alkaline environment of the phase transfer process. Therefore, after the phase transfer using PMAO, the HNPs can disperse in aqueous media (**Figure** **4**a).^[^ ^27^ ^]^

To examine the stability of the Fe_3_O_4_@Au HNPs after phase transfer, we employed dynamic light scattering to measure their hydrodynamic size. The results in Figure 4b show that all hybrid samples exhibit a single peak with a narrow base width, suggesting that the HNPs coated with PMAO are relatively uniform. The hydrodynamic sizes of the hybrid samples FA40, FA50, and FA60 increased to 31, 37, and 48 nm, respectively, corresponding to an increase in the Au shell thickness from 4.4 to 9.1 nm (TEM method). Figures 4c–e shows the TEM images of the PMAO‐coated Fe_3_O_4_@Au HNPs. The hybrid NP remained well dispersed after PMAO coating without any agglomeration.

### 2.4 Contrast Enhancement In Vitro CT Imaging of Fe_3_O_4_@Au HNPs

Based on the principle of X‐ray absorption by elements with high atomic numbers, especially gold, we have investigated the potential of enhancing CT image contrast using Fe_3_O_4_@Au HNPs. By adjusting the thickness of the gold shell, we have evaluated the efficiency of these NPs in X‐ray absorption. To quantify the absorption capacity, we used eFilm workstation software (Merge Healthcare, Chicago, IL, USA) to analyze the Hounsfield units (HU) values in the regions of interest of the CT images.

**Figure 5:**
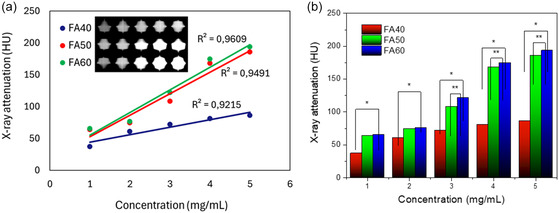
a) CT images of Fe_3_O_4_@Au NPs with varying shell thicknesses at different concentrations and corresponding linear plot of Hounsfield units, and b) corresponding X‐ray attenuation intensity.

**Figure** **5** clearly illustrates the relationship between concentration and X‐ray attenuation of Fe_3_O_4_@Au NPs with different gold shell thicknesses, corresponding to FA40 (Au shell thickness ≈4.4 nm), FA50 (Au shell thickness ≈6.6 nm), and FA60 (Au shell thickness ≈9.1 nm) at various concentrations. The results show a strong linear correlation between NP concentration and HU signal intensity (*R* ^2^ > 0.92), confirming their potential for enhancing image contrast. Further analysis of the effect of gold shell thickness revealed a significant increase in signal intensity as the shell thickness increased from 4.4 nm (FA40) to 9.1 nm (FA60). The results are consistent with the X‐ray absorption mechanism of the material, where more gold atoms lead to higher absorption. The results also align with the previous studies on spherical Au NPs and other reports on Fe_3_O_4_@Au HNPs.^[^ ^28^, ^29^, ^30^, ^31^, ^32^, ^33^ ^]^ To quantify the X‐ray attenuation, we converted the signal intensity into HU by calibrating with the values of air (−1000 HU) and water (0 HU). Specifically, the results showed that at a concentration of 3 mg mL^−1^, the FA60 sample had the highest HU value (122.12 HU), followed by FA50 (108.58 HU) and FA40 (72.31 HU). However, this enhancement tended to slow down at thicker shells, suggesting a certain saturation point. Based on the results, the Fe_3_O_4_@Au HNPs (FA50)with an average gold shell thickness of about 6–7 nm improved the contrast of X‐ray CT images.

Based on the promising results regarding X‐ray absorption capacity and high stability in aqueous solution, we conducted in vivo experiments to evaluate the effectiveness of FA50 NPs as a contrast agent for CT imaging.

**Figure 6:**
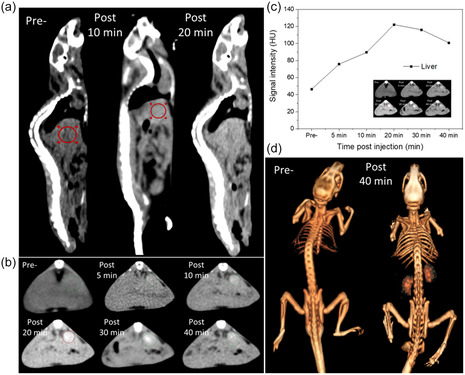
a,d) CT images of normal mouse pre‐ and postsystematic administration of Fe_3_O_4_@Au. b) Time evolutional in vivo CT imaging of mouse using hybrid nanofluid Fe_3_O_4_@Au over time: pre‐ and 5, 10, 20, 30, 40 min postinjection (circle) sites and c) contrast enhancement of the ROI in respect of HU values pre‐ and postcontrast.

After injecting FA50 intravenously into mice, CT scanning was performed. **Figure** **6**a shows in vivo CT images with a voltage of 80 kV of the mouse body before and after injection. The mouse body image brightened after FA50 injection (Figure 6a). As previously reported, NPs in general with a diameter greater than 8 nm will be captured by Kupffer cells of the reticuloendothelial system in living organisms and lead to accumulation in the liver.^[^ ^30^, ^34^, ^35^, ^36^, ^37^ ^]^ The liver region (indicated by the ROI, red circle) brightened significantly after injection. Figure 6b shows the CT image with signal intensity HU in the liver tissue before and after injection at different times. The bright signal intensity increased after using the contrast agent, with a significant difference in the body's signal intensity of the mouse after 5, 10, and 20 min of injection compared to before injection, with the signal intensity increasing from 46.5 HU to 75.8 HU, 89.5 HU, and 122.1 HU (Figure 6b,c). For CT images, we achieved a uniform contrast enhancement throughout the entire liver parenchyma after 5 min of injection. The intensity gradually enhanced within 20 min (Figure 6b,c). This result demonstrates the ability of NPs to accumulate in the liver, a phenomenon previously reported for NPs of similar size.^[^ ^28^ ^]^ The X‐ray signal intensity (HU) in both blood vessels and liver tissue was significantly enhanced compared to the image before scanning, providing strong evidence for the accumulation of Fe_3_O_4_@Au HNPs in the liver. However, the HU signal intensity intentionally decreased with time after 20 min (Figure 6c). This signal enhancement is maintained for a certain period before gradually decreasing, indicating the body's clearance process. This result confirms the effectiveness of FA50 as a CT contrast agent and provides essential information on the pharmacokinetics of NP distribution and excretion in the body. This result is consistent with the 3D CT image showing that after 30 min, the mouse kidney image had a signal (Figure 6d).

## 3 Conclusion

This study focuses on developing Fe_3_O_4_@Au HNPs for potential applications in medical imaging. Using a thermal decomposition method, we successfully synthesized monodispersed magnetite NPs (Fe_3_O_4_ NPs) with an average size of 9.4 ± 0.8 nm as seeds. Subsequently, a gold shell was coated onto the surface of the Fe_3_O_4_ core, forming a stable core–shell structure. The thickness of the gold shell can be flexibly adjusted, allowing for the optimization of X‐ray absorption. Evidenced by in vitro and in vivo experiments using a mouse model, we have demonstrated that Fe_3_O_4_@Au HNPs can significantly enhance CT image contrast, especially in liver tissues. Additionally, the study provides detailed information on the distribution and excretion processes of the NPs within the body. These results open up broad prospects for the application of Fe_3_O_4_@Au HNPs in the field of medical imaging.

## Conflict of Interest

The authors declare no conflict of interest.

## Author Contributions

**Nguyen Thi Ngoc Linh**: data curation (equal); formal analysis (equal); methodology (equal); project administration (equal); supervision (equal); writing—original draft (equal); writing—review and editing (equal). **Nguyen Hoa Du**: formal analysis (equal); methodology (equal); writing—original draft (equal); writing—review and editing (equal). **Ngo Thanh Dung**: data curation (equal); formal analysis (equal); funding acquisition (equal); project administration (equal); software (equal); supervision (equal). **Le Thi Thanh Tam**: data curation (equal); formal analysis (equal); methodology (equal). **Pham Hong Nam**: formal analysis (equal); methodology (equal); software (equal). **Phan Thi Hong Tuyet**: data curation (equal); formal analysis (equal); software (equal). **Le Trong Lu**: data curation (equal); formal analysis (equal); funding acquisition (equal); project administration (equal); writing—review and editing (equal). **Le The Tam**: data curation (equal); formal analysis (equal); investigation (equal); project administration (equal); supervision (equal); writing—original draft (equal); writing—review and editing (equal).

## Supporting information

Supplementary Material

## Data Availability

The data that support the findings of this study are available on request from the corresponding author. The data are not publicly available due to privacy or ethical restrictions.
