# Supplementary material for: Synthesis of Fe3O4@Au Core–Shell Nanoparticles with Varying Thicknesses for Application in Computed Tomography Imaging
Source: ChemistryOpen. 2025 Apr 21;14(10):e202500166. doi: 10.1002/open.202500166 (PMC12518033; doi:10.1002/open.202500166)
Supplement: Supplementary file 1 — Supplementary Material [file OPEN-14-e202500166-s001.pdf]

Supporting Information

Synthesis of Fe_3O_4@Au hybrid nanoparticles with varying thicknesses for application in computed tomography (CT) imaging

Nguyen Thi Ngoc Linh,[a] Nguyen Hoa Du,[b] Ngo Thanh Dung,[c] Le Thi Thanh Tam,[c,d] Pham Hong Nam,[c,d] Phan Thi Hong Tuyet,[b] Le Trong Lu [c,d] and Le The Tam,*[b]

Table of contents

1. Experimental Section……………………………………………………….03

2. References………………………………………………………………….06

Experimental Section

Materials: All chemicals used in the experiments were purchased from Sigma-Aldrich (Singapore), including iron(III) acetylacetonate (Fe(acac)$_3$), $FeCl_2.4H_2O$, gold(III) chloride trihydrate ($HAuCl_4.3H_2O$), sodium oleate (SOA), 1-octadecanol (OCD-ol), poly (maleic anhydride-alt-1-octadecene) (PMAO), 1-octadecene (ODE), dibenzyl ether (DBE), sodium hydroxide (NaOH), chloroform ($CHCl_3$), absolute ethanol (C_2H_5OH) and n-hexane (C_6H_{14}).

Preparation

Synthesis of Fe_3O_4@Au HNPs: The In this study, Fe_3O_4@Au HNPs were synthesized in two steps illustrated in Fig. 1:

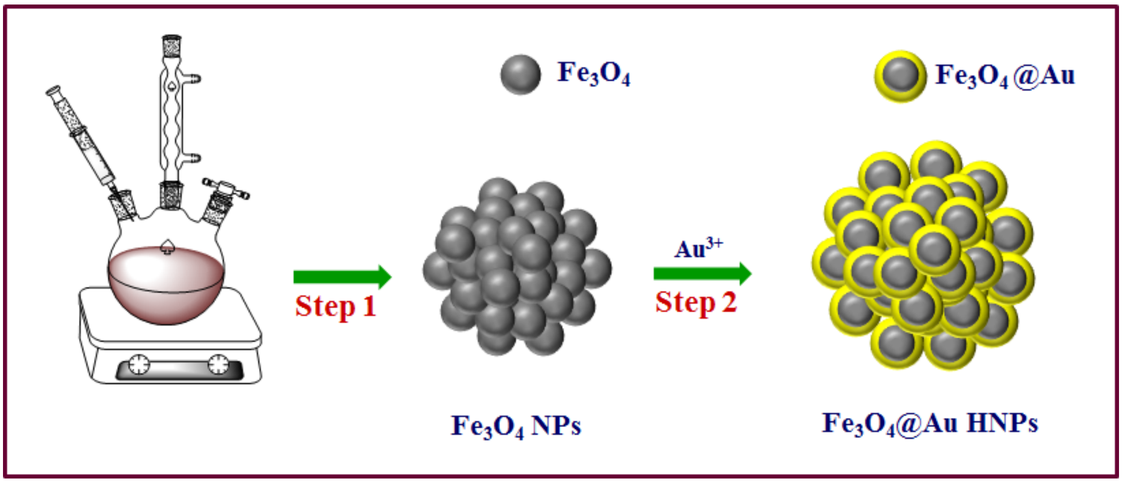

Scheme S1. Schematic illustration of the synthesis of Fe_3O_4@Au HNPs

In the initial stage, Fe_3O_4 NPs were synthesized via thermal decomposition in an organic solvent at high temperatures, following our previous work [1]. A mixture, which contains $Fe(acac)_3$ (120 mM), $FeCl_2.4H_2O$ (60 mM), SOA (720 mM), and OCD-ol (300 mM) in 40 mL ODE, was stirred in a three-neck flask under a nitrogen atmosphere. The reaction mixture was stirred magnetically at room temperature for 30 minutes to remove air. The reaction temperature was slowly increased to 100 °C and stirred magnetically for another 30 minutes to remove residual water. Subsequently, the reaction temperature was increased to 200 °C and refluxed for 30 minutes to form iron intermediate complexes. Finally, the reaction was heated to 270 - 315 °C and refluxed for 60 minutes. After a slow cooling process to room temperature, the product was obtained and washed with ethanol and n-hexane solvents, and Fe_3O_4 NPs were collected using a magnetic bar.

The next step involved the formation of Fe_3O_4@Au HNPs using the seeded-growth method [2], employing previously synthesized Fe_3O_4 NPs as seeds. Briefly, 5 mL of n-hexane containing 100 mg of Fe_3O_4 NPs, OCD-ol, SOA and $HAuCl_4.3H_2O$ were added to 50 mL of DBE in a three-neck flask. The final concentrations of OCD-ol and SOA are 15 and 100 mM, respectively. The concentration of $HAuCl_4.3H_2O$ varied in the range of 30, 40, 50, and 60 mM in the samples, correspondingly labelled FA30, FA40, FA50, and FA60. The reaction mixture was stirred magnetically at room temperature for 30 minutes, then heated to 80 °C and held at this temperature for 30 minutes to remove n-hexane. Finally, the reaction temperature was increased to 180 °C and refluxed for 60 minutes. A continuous nitrogen gas

flows through the entire process. The product obtained after the reaction was gradually cooled to room temperature and washed with ethanol and n-hexane solvents, similar to the washing process for Fe_3O_4 NPs, and a magnetic bar was used to remove free Au NPs, yielding the Fe_3O_4@Au HNPs. For comparison, Au NPs were synthesized similarly but without using Fe_3O_4 seeds.

Phase transfer of Fe_3O_4@Au HNPs into water

50 mg of Fe_3O_4@Au HNPs was well-dispersed under sonication in 1 mL of chloroform, forming solution 1. Solution 2 contained 1 g of poly(maleic anhydride-alt-1-octadecene) (PMAO) homogeneously dispersed in 10 mL of chloroform. Gradually add solution 1 to solution 2 until the particles were evenly dispersed. The resulting product was left at room temperature and stirred magnetically for all the chloroform to evaporate. 12 mL of 1M NaOH solution was added while stirring to obtain a homogeneous solution. The product was continuously washed with distilled water to reach pH 7, yielding water-dispersible Fe_3O_4@Au HNPs.

Methods of characterization

Characterization of nanoparticles

The morphology and particle size of the synthesized material were determined using transmission electron microscopy (TEM) on a JEMJEOL-1010 instrument (Japan). The particle size distribution was plotted using Origin software. High-resolution transmission electron microscopy (HR-TEM) on a JEM 2100, Joel instrument was used to obtain detailed images of the material's structure. The crystal structure of the material was determined by X-ray diffraction (XRD) on a Siemens D5005 instrument using Cu-kα radiation ($\lambda = 1.5406$ Å). The chemical composition of the material was determined by energy-dispersive X-ray spectroscopy (EDX) on an SEM-EDX instrument (Jeol 6490 - JED 2300). The UV-Vis absorption spectrum of the material was analyzed using a Jasco V-670 spectrophotometer (Japan). The magnetic properties of the material at room temperature were measured using a vibrating sample magnetometer (VSM) with a field of up to 10 kOe. Dynamic light scattering (DLS) measurements and colloidal stability were examined on a Zetasizer instrument (Malvern, UK).

In-Vitro CT Samples Preparation

To Imaging wells were prepared using agarose gel to immobilize the samples to investigate the influence of Fe_3O_4@Au hybrid nanoparticle size with varying gold shell thickness on CT contrast. The preparation is as follows: (i) dissolving 1 g of agarose in 100 mL of distilled water at 80 °C, (ii) dispersing Fe_3O_4@Au hybrid nanoparticle samples with different sizes in water at various concentrations (1, 2, 3, 4, and 5 mg/mL), (iii) placing the Fe_3O_4@Au hybrid nanoparticle samples in 2 mL wells, and positioning in a custom-designed scanning holder. The CT performance was on a 128-Somatom Perspective CT scanner (Siemens, Germany) with the following imaging parameters: source voltage 100 kV, 80 mA, slice thickness 0.6 mm, field of view (DFOV) x-y 278 x 295 mm^2, matrix size 541 x 510. The digital CT images in a standard display program supported by eFilm workstation software (Merge Healthcare, Chicago, IL, USA) can evaluate X-ray attenuation. A uniform region of interest (ROI) (cm^2) was selected on the obtained CT images for each sample. For each set of measurement conditions, the contrast intensity enhancement of the material was determined in Hounsfield units (HU).

Statistical analysis

The significance of the experimental data was assessed by one-way ANOVA with p-values of 0.05 and 0.01 were considered significant.

References

1. N. T. N. Linh, N. T. Dung, L. T. T. Tam, L. T. Tam, N. P. Hung, N. D. Vinh, N. T. Ha, P. H. Nam, L. V. Thanh, N. V. Dong, L. G. Nam, N. V. Dang, N. X. Phuc, L. D. Tung, N. T. K. Thanh, L. T. Lu, *New J. Chem.* **2023**, *47*, 4052–4067.
2. J. Jiang, H. Gu, H. Shao, E. Devlin, G. C. Papaefthymiou, J. Y. Ying, *Adv. Mater.* **2008**, *20*, 4403–4407.
